# Airway Management of a Patient With Tracheoesophageal Fistula and Tracheal Stent

**DOI:** 10.7759/cureus.30524

**Published:** 2022-10-20

**Authors:** Usmar Ashraf, Omer Farooq, Allah Ditta Ashfaq, Fahad Butt, Shahid Khattak

**Affiliations:** 1 Anesthesia and Pain Management, Shaukat Khanum Memorial Cancer Hospital and Research Centre, Lahore, PAK; 2 Anesthesia and Pain Management, Shaukat Khanum Memorial Cancer Hospital and Research Centre, lahore, PAK; 3 Anesthesia and Critical Care, Shaukat Khanum Memorial Cancer Hospital and Research Centre, Lahore, PAK; 4 Anesthesia, Shaukat Khanum Memorial Cancer Hospital and Research Centre, Lahore, PAK; 5 Surgical Oncology, Shaukat Khanum Memorial Cancer Hospital and Research Centre, Lahore, PAK

**Keywords:** tracheal stent, squamous cell carcinoma, endobronchial intubation, tracheal stenosis, one lung ventilation, tracheo-esophageal fistula

## Abstract

A 69-year-old male patient, a known case of squamous cell carcinoma of the esophagus on palliative care and Do Not Attempt Resuscitation (DNAR) status, presented for urgent laparoscopic gastrostomy tube insertion under general anesthesia. The patient had developed an iatrogenic tracheoesophageal fistula (TEF) because of the tracheal stent, which was placed for tracheal stenosis. A preoperative assessment was done, and a plan of airway management via one-lung ventilation (OLV) through an endobronchial tube was devised by the anesthesia team and discussed with the surgery team.

The airway was secured via asleep fiberoptic right endobronchial intubation using a microlaryngeal tube (MLT) size 6 since there was uncertainty regarding adequate patency of the airway due to the invasion by the tumor and the presence of the stent. The patient remained hemodynamically stable. After surgical incision and insufflation of CO_2_ in the abdominal cavity, the patient’s airway pressures were increased and we were unable to deliver adequate tidal volumes. Surgery was stopped; the presence of a kink in the circuit or endotracheal tube (ETT), the possibility of laryngospasm/bronchospasm, and pneumothorax were ruled out. Fiberoptic bronchoscopy (FOB) revealed that the endobronchial tube was abutting the secondary carina. We pulled the MLT by 2 cm. The rest of the procedure was uneventful and we extubated the patient at the end of the procedure under vision using a fiber optic bronchoscope. The patient was discharged after two days of stay in the hospital.

Our patient with TEF and tracheal stent posed a significant challenge for airway management. A thorough plan was drawn up and a team briefing was done. Perioperatively, the difficulty in ventilation was identified, and various other etiologies were ruled out with the successful identification and management of the problem.

## Introduction

Tracheoesophageal fistula (TEF) is a congenital abnormality seen in infants or acquired later in the adult population [1]. It is a serious complication that can lead to morbidity and mortality [1-4]. Patency between the airway and the upper gastrointestinal (GI) tract bypasses the normal protection offered by the laryngeal reflexes. As a consequence, patients are at an increased risk of aspiration and an airway leak during positive pressure ventilation [1-4]. Upper GI surgery poses an increased risk of aspiration due to pressure alteration and manipulation across the gastro-esophageal sphincter. The presence of a tracheal stent in the upper trachea could lead to the narrowing of the airway. Also, the chances of stent dislodgement are higher during airway manipulation, which may result in catastrophic consequences.

Anesthetic options available for upper GI surgery are general and regional anesthesia. However, the presence of a tracheal stent, formation of a TEF, and requirement of laparoscopic upper GI surgery necessitate the employment of a general anesthetic technique. General anesthesia in such a setting is challenging and there are various options and modalities available, such as the use of fiberoptic bronchoscopy (FOB), conventional or video laryngoscopy, one-lung ventilation (OLV), and deep versus awake extubation. The use of FOB helps in complicated airway management and is becoming an essential component of the management strategies for difficult airways.

We present a case of successful general anesthetic management with FOB guidance in a patient requiring urgent upper GI laparoscopic surgery, who had advanced esophageal cancer, acquired TEF, and tracheal stent in situ.

## Case presentation

A 69-year-old male patient, weighing 56 kilograms, 164 centimeters in height with a BMI of 20.9, a known case of squamous cell carcinoma of the distal esophagus, was scheduled to undergo urgent feeding gastrostomy tube insertion under laparoscopic guidance by the surgical team. The urgency was determined by the presence of TEF and dysphagia due to the tumor itself, which had led to significant weight loss. Feeding gastrostomy tube insertion could not be undertaken through radiological guidance as the patient's colon was covering the stomach anteriorly and access to the stomach could not be attained.

The patient had been diagnosed with squamous cell carcinoma of the esophagus in 2012. The extent of the tumor on esophagoscopy was 30-38 cm from incisors. The patient had received chemoradiotherapy, but the tumor had relapsed in 2017 and he had subsequently undergone a second round of chemoradiotherapy. In 2020, the patient had presented with progressive dysphagia, more to solids than liquids. The tumor had been infiltrating the trachea just above the carina causing tracheal stenosis. Tracheal stenting had been done to relieve stenosis in 2020. The patient had developed TEF post tracheal stenting that was diagnosed on a CT scan done in April 2021.

**Figure 1:**
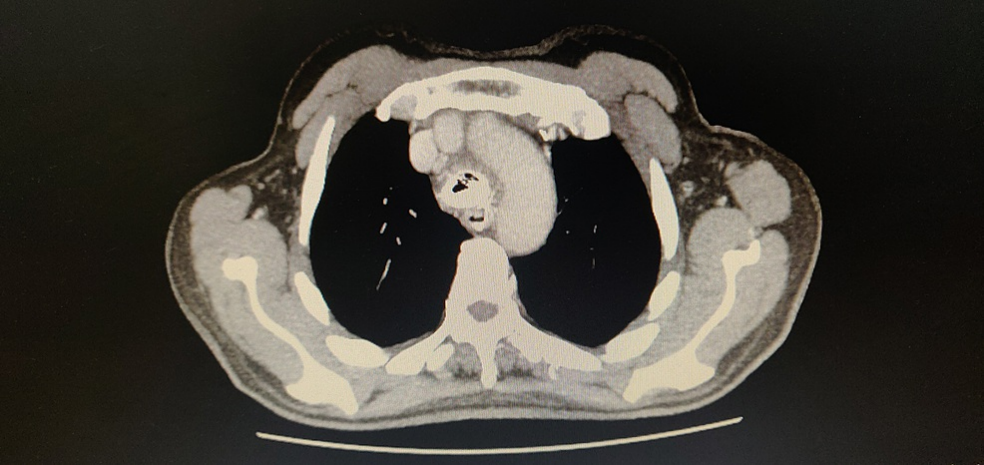
Preoperative CT chest showing fistulous communication between trachea and esophagus caused by tracheal stent - image 1 CT: computed tomography

**Figure 2:**
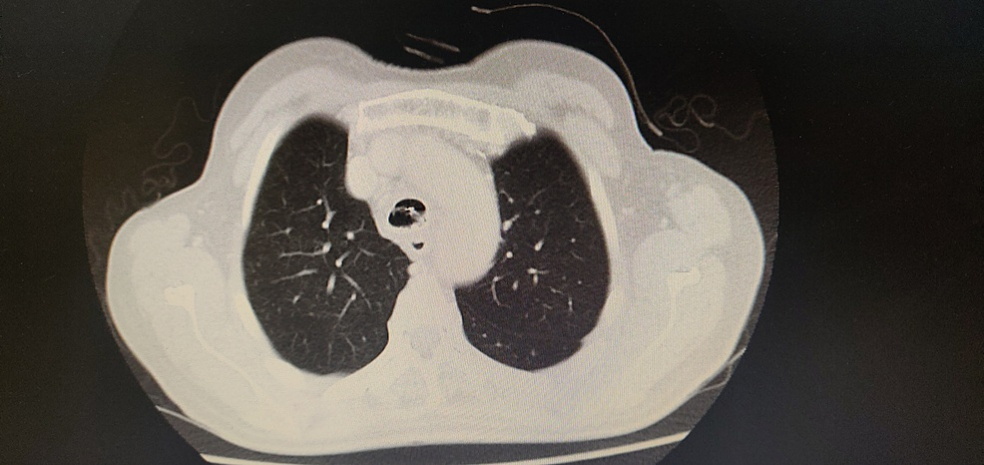
Preoperative CT chest showing fistulous communication between trachea and esophagus caused by tracheal stent - image 2 CT: computed tomography

The patient developed dysphagia and complained of cough on trying to swallow solids and liquids alike. CT scan after oral contrast revealed a fistula between the esophagus and trachea just above the carina as the contrast was seen in bilateral main and segmental bronchi (Figures 1, 2). The patient was currently on palliative care treatment and had a Do Not Attempt Resuscitation (DNAR) order in place because of metastatic disease.

A preoperative anesthesia assessment was done. The patient had no associated comorbidities, and there was no history of drug allergies. A thorough airway assessment was performed and it was labeled as an anticipated difficult airway.

In view of the patient’s condition, a plan of general anesthesia with standard monitoring and a backup of invasive monitoring was made. Asleep fiber optic-assisted bronchial intubation was planned to secure the airway. The patient and his relatives were counseled about the risks and benefits and high-risk informed consent was obtained. Theatre staff including the anesthesia team, surgical team, and nurses were briefed about the airway management of the patient.

Before shifting the patient to the operating theatre, a difficult airway trolley (DAT) was made accessible. FOB and other equipment required were made available. The team discussed the Difficult Airway Society (DAS) algorithm [5] and made the guidelines accessible in the theatre. About 30 minutes before the induction, intravenous glycopyrrolate 0.2 mg was given to reduce the oropharyngeal secretions. In the operating room, after applying the standard AAGBI (Association of Anesthetists of Great Britain and Northern Ireland) monitoring [6], preoxygenation with 100% FiO_2_ was initiated. General anesthesia induction was done with midazolam 3 mg, fentanyl 100 micrograms, and propofol 200 mg in titrated doses. After establishing successful bag-mask ventilation with peak pressures of less than 20 cm of H_2_O, atracurium 30 mg was given. Bag-mask ventilation was continued for three more minutes. A cuffed microlaryngeal tube (MLT) of size 6.0 was railroaded on a FOB and was introduced through the oral cavity. After reaching the floor of the mouth, a jaw thrust maneuver was done by the anesthesia assistant. Vocal cords were visualized and FOB was introduced into the central airway. The tracheal stent was visualized in place; it was intact and patent with fibrotic tissue seen around it. TEF and tracheal stent were bypassed and an attempt was made to introduce MLT via FOB in the left main bronchus. This attempt was unsuccessful as the acute angulation of the left main bronchus did not permit the safe passage of MLT. It was then decided to intubate the right main bronchus under FOB guidance. MLT was advanced toward the right main bronchus. MLT cuff was inflated with air and endobronchial intubation was confirmed with right-sided chest rise, bilateral auscultation, and capnography. Positive pressure ventilation with pressure control ventilation mode was started. Tidal volumes of around 400 ml were achieved by setting inspiratory pressure of 12 mmHg, positive end-expiratory pressure (PEEP) of 4 mmHg, and respiratory rate of 12 breaths per minute. The flow-volume loop was consistently monitored for any abnormality.

Initially, the patient was ventilated with pure oxygen. FiO_2_ was reduced to 0.5 by gradually mixing air with oxygen. The patient remained hemodynamically stable throughout this process.

Anesthesia was maintained with sevoflurane. End-tidal sevoflurane concentration of 2.1-2.3% was maintained. A surgical timeout was performed and an umbilical incision was made to insert a laparoscopic port for the camera and CO_2_ insufflation to achieve pneumoperitoneum. Morphine 2 mg was given before the surgical incision. Within the first few minutes of CO_2_ insufflation, we were unable to achieve the required tidal volumes and there was an increase in peak airway pressures. FiO_2_ was increased to 1.0 immediately and the surgical team was alerted and advised against any surgical manipulation until the ventilation issue was resolved. The anesthesia circuit and MLT were checked for any obstruction. Auscultation revealed absent breath sounds on the right side. The suction catheter was introduced through MLT to clear out possible secretions or mucous plugs at the end of MLT. There was some resistance felt in the passage of the suction catheter. All these efforts provided no respite. Meanwhile, the patient’s oxygen saturation (SpO_2_) started to fall; the lowest SpO_2_ reading recorded was 85%. FOB was introduced through MLT and it was observed that MLT was abutting the secondary carina inside the right main bronchus. MLT was pulled out by 2 cm under vision. Manual ventilation was reestablished with no difficulty and positive pressure ventilation resumed with the ventilator settings of inspiratory pressure of 12 mmHg, PEEP of 4 mmHg, respiratory rate of 12 breaths per minute, and FiO_2_ of 100%. FiO_2_ was again decreased to 50%. The surgical team was updated and the procedure resumed. The patient remained hemodynamically stable for the rest of the procedure. Paracetamol 1 G was given for analgesia. Dexamethasone 8 mg and ondansetron 4 mg were given as antiemetic prophylaxis.

The total duration of the surgery was 90 minutes. At the end of the surgical procedure, MLT was withdrawn under FOB guidance into the distal trachea above the carina to re-inflate the collapsed left lung. FOB was introduced in the left bronchus to see if there were any secretions or plugs. A gentle increase in inspiratory pressure facilitated the re-expansion of the collapsed left lung, which was confirmed on auscultation. The patient regained spontaneous breathing and was reversed with neostigmine 2.5 mg and glycopyrrolate 0.5 mg as guided by neuromuscular monitoring via nerve stimulator and the train-of-four count. FOB was again introduced through MLT. The patient was extubated under vision. The tracheal stent was visualized and no abnormality could be seen. There were no added secretions or evidence of aspiration in both the main bronchi and trachea. After extubation, the patient maintained his saturation above 94% on 4 L of oxygen while breathing spontaneously with tidal volumes of 400-500 ml at a rate of 16-18 breaths per minute. The patient was monitored inside the operating room for 15 minutes and then shifted to the post-anesthesia care unit (PACU) for an extended stay in recovery. The patient remained hemodynamically stable and was transferred to the surgical floor after achieving a modified Aldrete score (MAS) of 10. The patient remained stable during the remainder of his hospital stay and was discharged after two days with an unremarkable recovery.

## Discussion

The case we presented was a challenging one as there were multi-faceted issues that required careful consideration in order to achieve the best possible outcome. The issues faced were as follows: the presence of a distal TEF, a tracheal stent in situ, poor nutritional status, and advanced disease because of which the patient was placed on DNAR status.

Acquired TEF is a very rare entity. While TEF is a complication rarely seen in adults, due to its risks of pulmonary complications, it has a very high mortality rate [7]. Tracheal injury often occurs as a result of trauma, vascular compression of the endotracheal tube (ETT) or tracheostomy tube cuff on the tracheal wall, or malignancy [1]. Over the last three decades, the etiology of acquired TEF has evolved. Iatrogenic, malignant, and traumatic causes have now superseded infection, formerly the predominant etiology of acquired TEF [2].

Malignancy is the most common cause of acquired TEF cases. Of these, 77% are attributable to esophageal tumors; 16% are secondary to pulmonary primaries. Esophageal tumors pose the greatest risk with an incidence of 4.5% [2-4]. The TEF forms as a result of necrosis and tissue breakdown. This is secondary to tumor enlargement and invasion but may be exacerbated by radiotherapy and chemotherapy. Untreated TEFs result in repeated airway soiling with rapid progressive pulmonary sepsis and death. The median survival from the time of diagnosis is one to six weeks [4].

Distal TEF poses an additional challenge for controlled ventilation as it is difficult to isolate the fistula without undertaking endobronchial intubation to bypass the site of the fistula and prevent inflation of the GI tract with positive airway pressure delivered by the ventilator [8]. If the tip of the ETT lies above the TEF, gastric dilatation and aspiration can occur. Intubating the lumen of a large TEF with failure to ventilate is a major concern. OLV poses risks of hypoxemia, iatrogenic lung injury, lung collapse, difficulty in ventilation, pneumothorax, and tracheal/bronchial injury [8-9].

The presence of a tracheal stent in an upper airway can lead to airway narrowing, fistula formation, and dislodgement during airway maneuvering [10]. This poses an anesthetic challenge as these complications could lead to catastrophic consequences. Tracheal stents are placed to maintain the patency of the trachea in order to prevent the collapse of the trachea. Hence tracheal stents play a vital role in sustaining and improving the quality of life in advanced cancer patients.

In order to deal with these risks, a detailed preoperative anesthesia assessment should be undertaken. Comorbid conditions should be optimized before surgery. The size and site of the TEF lesion must be noted carefully as this may dictate the anesthetic approach. Also, the place of the tracheal stent on radiological imaging should be noted. If there are any doubts, a discussion with radiology colleagues should be sought. A comprehensive anesthetic plan should be made and discussed with the members of the anesthesia and surgery teams.

We undertook a thorough history, examination, and investigation for the procedure. A detailed discussion with the surgical team was conducted and the challenges posed by anesthetic management were considered. The plan was made to proceed with laparoscopic surgery, but it was agreed that in case there were problems with patient ventilation, the procedure could be converted to open or even abandoned.

Antisialagogues such as glycopyrrolate are preferred to be used as they help in reducing salivary and respiratory secretions and eventually ease intubation. For a patient with cardiac problems, cautious use of glycopyrrolate is advised as it could cause tachycardia. In our patient, glycopyrrolate was given about 30 minutes before the intubation was done. We did not encounter many secretions during the procedure. The patient was taking proton pump inhibitors (omeprazole 20 mg) twice daily. On the morning of surgery, omeprazole 40 mg IV was given in order to minimize the risk of aspiration. Glycopyrrolate is also preferred to be used for reducing the orotracheal secretions.

Anesthetic management during TEF repair includes challenges of possible difficult intubation, sharing the airway with the surgical team, troubles during apnea periods, and a large amount of leakage during ventilation, while providing a good anesthetic depth so the instrumentation does not cause issues. Preoxygenation with 100% FiO_2_ provides an additional safety net during intubation before the patient starts to desaturate. This improves the oxygen reservoir by denitrogenation [11] and helps in difficult airway situations. We pre-oxygenated our patient for three minutes and started the induction process once the ETO_2_ of more than 85% was achieved.

The induction of anesthesia could be done through intravenous or inhalational techniques. Caution should be taken before administrating muscle relaxants. Successful establishment of bag-mask ventilation before administering neuromuscular blockade would provide reassurance that the oxygenation could be maintained if the intubation turns out to be difficult.

We opted for IV induction rather than gas induction as with gas induction, it would have been difficult to gauge the adequacy of end-tidal volatile concentration due to the presence of TEF leading to a gas leak in the esophagus. The option of intubation was chosen ahead of the use of any supraglottic device as we had to secure the airway to prevent the risk of aspiration. An intermediate-acting muscle relaxant was used in order to achieve the best possible conditions to place the ETT in the right place after examining the tracheobronchial path.

In the presence of TEF, the patient could have spontaneous or controlled ventilation. The spontaneous mode could be used as it helps in avoiding positive pressure ventilation and thus reduces the chance of an air leak through the tracheobronchial tree. But adequacy of ventilation and depth of anesthesia maintenance through volatile agents could not be ensured. Rapid bypass of the TEF is the key to successful anesthetic management.

We chose controlled ventilation as the patient was undergoing laparoscopic upper GI surgery and it would have been difficult to maintain adequate tidal volumes on spontaneous ventilation in the presence of pneumoperitoneum. For proximally located TEFs, intubation can be done by a conventional method. ETT should bypass TEF and remain above the carina. Intubation in distally placed TEFs is tricky. Awake or sleep FOB intubation needs to be considered to bypass the fistula. Adequate placement of ETT to conduct single lung ventilation should be considered in these cases.

Once the TEF is bypassed by endotracheal intubation, positive pressure ventilation could be maintained with minimal risk of soiling and gastric dilatation. Anesthesia can be maintained with a volatile anesthetic agent or total intravenous anesthesia and muscle relaxation. The patient should be carefully monitored throughout the procedure for any signs of air leaks or ventilator problems. We used OLV in order to bypass the distal TEF and used control mode ventilation (CMV) to maintain adequate ventilation throughout the procedure. The course of ventilation remained uneventful barring one episode of raised peak pressure and temporary patient desaturation due to the migration of MLT distal to its original position as the intraabdominal pressure was increased.

Once the procedure was complete, it was essential to expand the other lung [8-9]. We ensured this by pulling the MLT into the distal trachea and by gently applying positive pressure in order to reduce leaks through TEF. Also, the left bronchus was visualized for any aspiration as it was not isolated during the procedure. We found no evidence of aspiration or spillage in the left bronchus.

Immediate extubation is the goal, thereby avoiding any postoperative ventilation [12]. Extubation is achieved with careful reversal of neuromuscular blocking agents. We used a neuromuscular stimulator to ensure that the residual effect of neuromuscular blockers had worn off and instituted neuromuscular blocker reversal in order to ensure adequate ventilation before extubation.

As airway manipulation could lead to dislodgement of the tracheal stent that could lead to catastrophic consequences, we ensured that while extubating the patient we visualized the stent in place and no damage was done to it. Also, after extubation, the patient was examined to rule out any subcutaneous emphysema. The patient did not have any signs or symptoms of subcutaneous emphysema.

In PACU, patients should be monitored carefully and discharged only by an anesthetist after evaluation and ensuring that the patient meets the discharge criteria. The patient was monitored in PACU for an extended period. The patient remained in PACU for three hours. He had a chest X-ray done in PACU to rule out any lung collapse, pneumothorax, or subcutaneous emphysema. Chest X-ray did not show any abnormality. The patient maintained his saturation with minimal oxygen requirements and did not develop any signs of emphysema or respiratory distress, and he was discharged to the surgical floor uneventfully.

## Conclusions

Anesthetic management of patients with TEF and tracheal stents poses a challenge to the anesthetic team. The use of FOB in such cases is very valuable as it could help in safe intubation, and at extubation, the tracheobronchial tree can be visualized to detect any trauma or inadvertent iatrogenic damage. This would help in the timely management of such patients who are already at a high risk of morbidity and mortality. Coordinated team effort is pivotal as all the team members involved in the care of high-risk patients need to be aware of the risks involved and the plan to deal with the anticipated difficulty if the need arises.
